# Influence of enhanced recovery after surgery programs on laparoscopy-assisted gastrectomy for gastric cancer: a systematic review and meta-analysis of randomized control trials

**DOI:** 10.1186/s12957-017-1271-8

**Published:** 2017-11-23

**Authors:** Zhengyan Li, Qian Wang, Bofei Li, Bin Bai, Qingchuan Zhao

**Affiliations:** Department of Surgery, Xijing Hospital of Digestive Diseases, The Fourth Military Medical University, No. 127 Changle West Road, Xian, 710032 China

**Keywords:** Fast track surgery, Enhanced recovery after surgery, Gastric cancer, Laparoscopy-assisted gastrectomy

## Abstract

**Background:**

This meta-analysis is aimed to evaluate the feasibility and safety of enhanced recovery after surgery (ERAS) programs in gastric cancer patients undergoing laparoscopy-assisted gastrectomy (LAG).

**Methods:**

We performed a meta-analysis of randomized control trials involving either enhanced recovery after surgery (ERAS)/fast track surgery (FTS) for patients underwent LAG. EMBASE, Pubmed, Web of science, and Cochrane Library were searched. Primary outcomes included the length of postoperative hospital stay, cost of hospitalization, postoperative complications, and readmission rate.

**Results:**

Five randomized control trials were eligible for analysis. There were 159 cases in FTS group and 156 cases in conventional care group. Compared with conventional care group, FTS group relates to shorter postoperative hospital stay (WMD − 2.16; 95% CI − 3.05 to − 1.26, *P* < 0.00001), less cost of hospitalization (WMD − 4.72; 95% CI − 6.88 to − 2.55, *P* < 0.00001), shorter time to first flatus (WMD − 9.72; 95% CI − 13.75 to − 5.81, *P* < 0.00001), lower level of C-reaction protein on postoperative days 3 or 4 (WMD − 19.66; 95% CI − 28.98 to − 10.34, *P* < 0.00001), higher level of albumin on postoperative day 4 (WMD 3.45; 95% CI 2.01 to 4.89, *P* < 0.00001), and postoperative day 7 (WMD 5.63; 95% CI 1.01 to 10.24, *P* = 0.02). Regarding postoperative complications, no significant differences were observed between FTS group and conventional care group (OR 0.63, 95% CI 0.37 to 1.09, *P* = 0.10). The readmission rate of FTS group was comparable to conventional care group (WMD 3.14; 95% CI 0.12 to 81.35, *P* = 0.49).

**Conclusions:**

Among patients undergoing LAG, FTS is associated with shorter postoperative hospital stay, rapid postoperative recovery, and decreased cost without increasing complications or readmission rate. The combined effects of the two methods could further accelerate clinical recovery of gastric cancer patients.

## Background

Gastric cancer is a worldwide health concern and is the second leading cause of cancer-related deaths in China [1]. In recent years, the use of laparoscopy-assisted gastrectomy (LAG) was developed and clinically implemented to treat gastric cancer with the advantage of better short-term outcomes [2–4]. Fast track surgery (FTS)/enhanced recovery after surgery (ERAS) was first introduced by Kehlet in the 1990s and has gained satisfactory curative effect in many fields of surgery [5–10]. ERAS guidelines have been established in many kinds of surgeries, such as colectomy, cystectomy, and stomach surgery. Recent meta-analyses have demonstrated that ERAS is safe and effective after laparoscopic hepatectomy and colorectal surgery [11–13]. Previous studies have demonstrated that ERAS could accelerate the postoperative recovery in open gastrectomy for gastric cancer [14–16]. However, the role of ERAS in LAG is still unclear. To date, several studies have reported the value of FTS in LAG [17–19]. But they are all based on single-center studies with small sample size which may influence the credibility of the results. Therefore, we conduct this meta-analysis to assess the effects of ERAS protocol in gastric cancer patients undergoing LAG.

## Methods

### Literature search

This meta-analysis was conducted on the basis of the preferred reporting items for systematic reviews and meta-analyses (PRISMA) guidelines [20]. EMBASE, Pubmed, Web of science, and Cochrane Library were searched from January 1995 to July 2017. Studies were limited to English and Chinese. We used the following key words: “fast track,” “enhanced recovery,” “FTS,” “ERAS,” “gastric cancer,” “laparoscopy-assisted gastrectomy,” and “laparoscopic gastrectomy.” Additionally, the reference lists of all included studies were also searched to retrieve related articles.

### Inclusion and exclusion criteria

Inclusion criteria categories included (1) patients undergoing LAG for gastric cancer (2) perioperative care using either ERAS or FTS protocols compared with standard or conventional care, (3) randomized controlled trials (RCTs)(4) clearly state the ERAS program, and at least one or more of the primary outcomes was reported. Studies were excluded if they (1) included less than 6 interventions items in the FTS group according to the ERAS guidelines [21] (2) unable to provide one of the primary outcome mentioned above.

### Outcome measures

The primary outcomes were the length of postoperative hospital stay, cost of hospitalization, postoperative complications, and readmission rate. The secondary outcomes were time to first flatus, level of C-reaction protein, albumin, and Interleukin-6.

### Quality assessment and data extraction

The quality of each included RCT was assessed according to the Cochrane methodology, which included the following evaluation domains: random sequence generation, allocation concealment, blinding of participants and personnel, blinding of outcome assessment, incomplete outcome data, selective reporting, and other biases [22]. The data was extracted from each eligible trial by two authors (Q Wang and Bin Bai). From each study, we extracted the general information of included studies, including the author, publication year, type of studies, sample size, surgery method, follow-up duration, and some other details.

### Statistical analysis

The data pooling was carried out using the Review Manager software (version 5.3, Nordic Cochrane Centre). The pooled results were expressed as the mean difference (MD) with 95% confidence interval (CI). Continuous variables were assessed using weighted mean difference (WMD). Dichotomous variables was analyzed using odds ratios (OR) and 95% CI.

Statistical heterogeneity among studies was evaluated by using the Cochran Q statistic and quantified by *I* ^2^ statistics. The random-effects model was used in this meta-analysis. Due to the limited number of studies (less than 10), the funnel plot was not performed to test the publication bias. *P* < 0.05 was considered statistically significant.

## Result

### Characteristics of trials

**Fig. 1:**
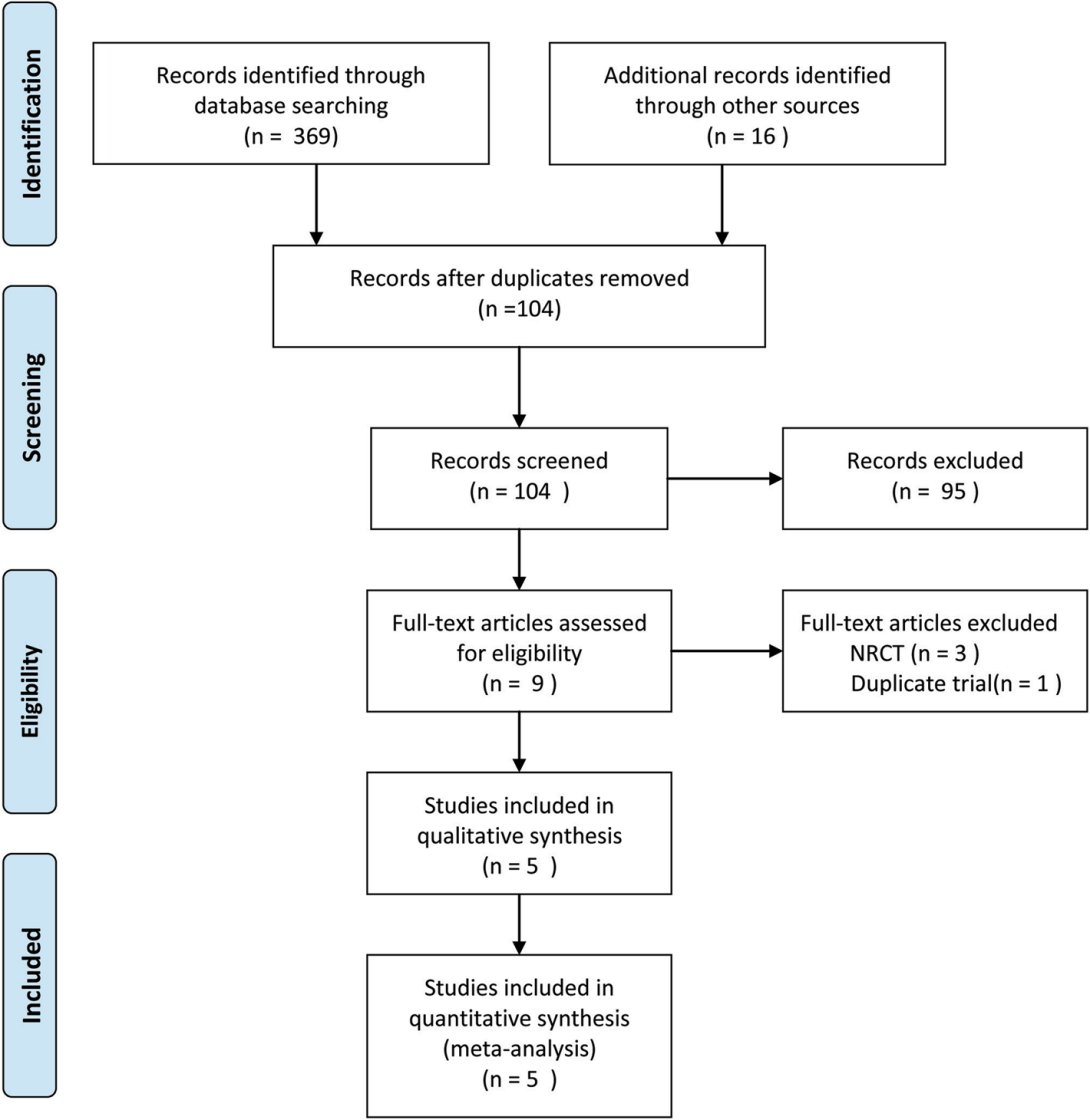
Flow chart for the selection of eligible studies

**Fig. 2:**
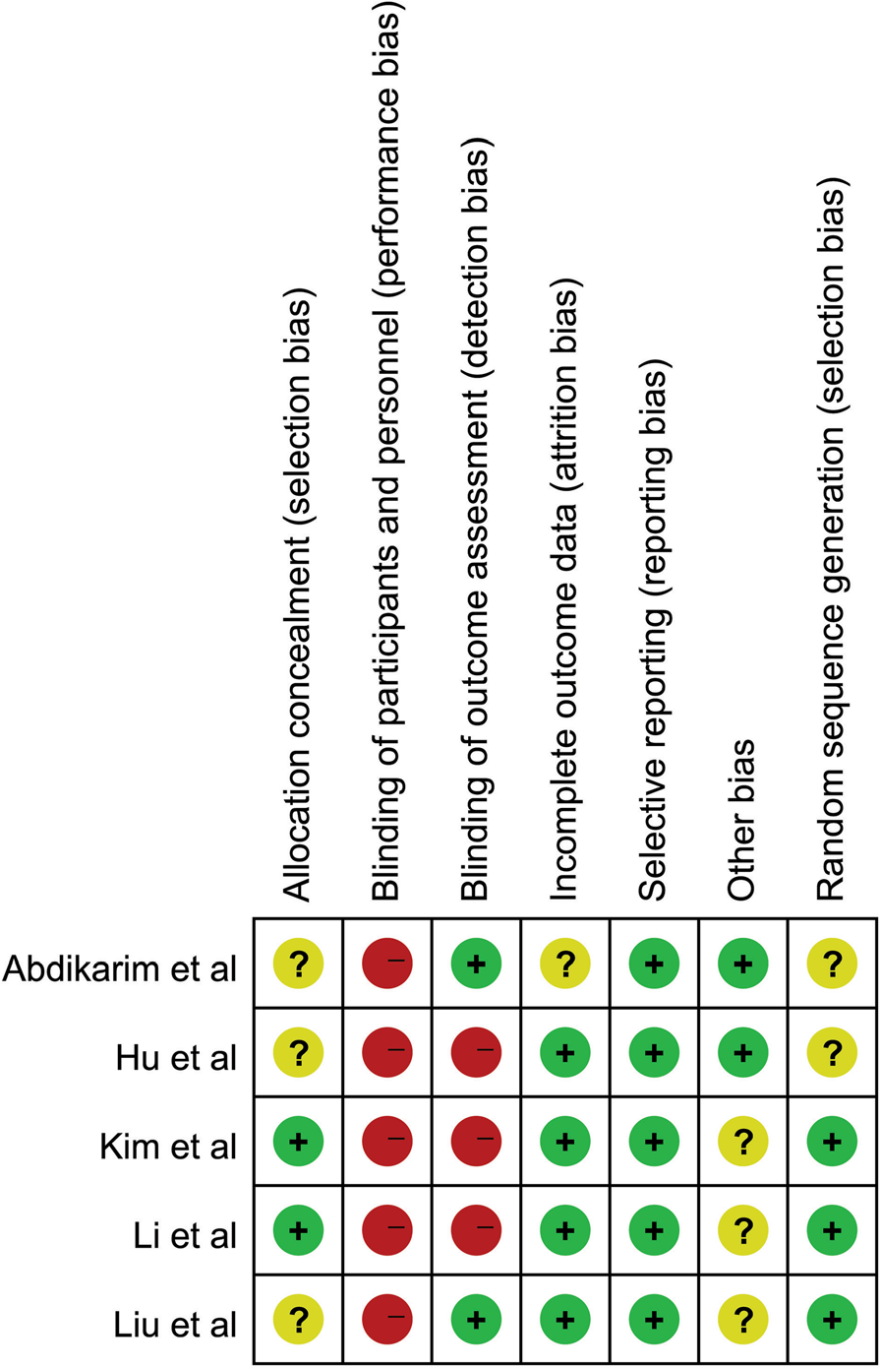
Risk of bias summary of all included studies. *Plus* low risk of bias, *minus* high risk of bias, *question mark* unclear risk of bias

Figure 1 summarizes the flow chart for the selection of eligible studies. Eventually, five studies [17–19, 23, 24] were considered eligible for this meta-analysis. In all, data from 315 patients were recorded, of which 159 in the FTS group and 156 in the conventional care group. All included studies were conducted in Asia (4 in China and 1 in Korea), and their studies were published between 2012 and 2016. Table 1 shows the general characteristics of included studies. The EARS items applied in the included studies are presented in Table 2. Regarding the methodological quality, all included studies showed low to moderate overall risks of bias (Fig. 2).

**Table 1:** Characteristics of included studies

Reference	Year	Type of study	Sample size		Surgery method	Outcomes	Follow-up duration	Age		BMI	
			FTS	CC				FTS	CC	FTS	CC
Kim et al.	2012	RCT	22	22	LAG	1.2.4.5.6.7	2 weeks	52.64 ± 11.5	57.45 ± 14.54	23.40 ± 3.17	23.77 ± 3.54
Hu et al.	2012	RCT	19	22	LAG	1.2.4.5.6.7	4 weeks	59 (49–71)	62.5(45–72)	22.94 ± 2.23	22.99 ± 2.24
Abdikarim et al.	2015	RCT	30	31	LAG	1.4.7	30 days	63 ± 12	62 ± 11	NR	NR
Li et al.	2016	RCT	67	60	LAG	1.4.7.8	2–21 months	72.9 ± 6.7	71.8 ± 8.0	21.1 ± 2.5	20.4 ± 2.3
Liu et al.	2016	RCT	21	21	LAG	1.2.3.4.5.7.8	NR	69.2 ± 5.1	70.3 ± 5.8	21.5 ± 2.0	21.9 ± 2.3

*RCT* randomized controlled trials, *FTS* fast track surgery, *CC* conventional care, *NR* not reported, *1* time to first flatus, *2* C-reaction protein, *3* interleukin-6, *4* length of postoperative hospital stay, *5* hospitalization expenditure, *6* readmission rate, *7* postoperative complications, *8* albumin

**Table 2:** EARS/FTS elements applied in the included studies

Element	Kim et al.	Hu et al.	Abdikarim et al.	Li et al.	Liu et al.
Preoperative counseling	√	√	√	√	√
Avoid preoperative bowel preparation	√	√	√	√	√
Preoperative carbohydrate loading	√	√	√	√	√
No pre-anesthetic medication	√	√	√	√	√
Prophylaxis against thromboembolism					
Antimicrobial prophylaxis					
Standard anesthetic			√		
Postoperative nausea and vomiting prophylaxis					
Minimal invasive surgery	√	√	√	√	√
Avoid nasogastric tube	√	√	√	√	√
Prevent hypothermia	√			√	
Perioperative fluid management			√		√
Avoid peritoneal drainage		√			
Early urinary removal	√	√	√	√	√
Postoperative analgesia	√		√	√	√
Early oral feeding	√	√	√	√	√
Early mobilization	√	√	√	√	√
Audit

### Meta-analysis results

#### Postoperative hospital stay and hospitalization expenditure

**Fig. 3:**
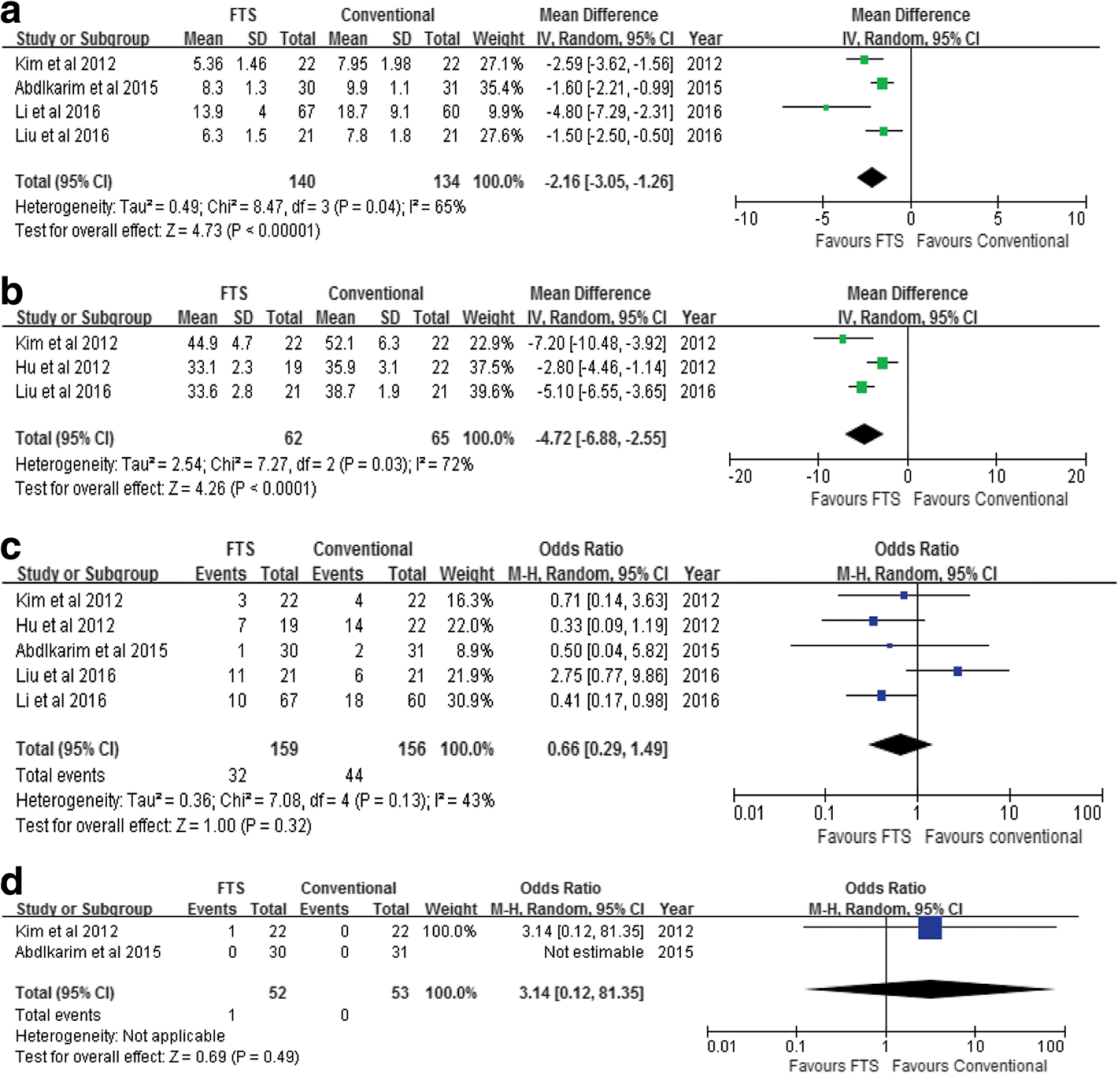
Meta-analyses of primary outcomes. **a** Postoperative hospital stay. **b** Cost of hospitalization. **c** Postoperative complications. **d** Readmission rate

The data of postoperative hospital stay could be obtained from four included studies. The result showed that FTS group is associated with a significant reduction in postoperative hospital stay when compared to the conventional care group (WMD − 2.16; 95% CI − 3.05 to − 1.26, *P* < 0.00001) (Fig. 3a). High heterogeneity was observed among the studies (*P* = 0.04, *I* ^2^ = 65%), and a random-effects model was used. Three included studies reported the cost of hospitalization. Results showed that FTS group had a less cost of hospitalization compared to the conventional care group (WMD − 4.72; 95% CI − 6.88 to − 2.55, *P* < 0.00001) (Fig. 3b).

#### Postoperative complications and readmission rate

Postoperative complications were described in all five studies. The results of this meta-analysis did not show a significant difference between the two groups (OR = 0.63, 95% CI 0.37 to 1.09, *P* = 0.10) (Fig. 3c). Two studies reported readmission rate of patients. No statistical difference was found between the two groups (WMD = 3.14; 95% CI 0.12 to 81.35; *P* = 0.49) (Fig. 3d).

#### Time to first flatus, ambulation time, and time to start diet

**Fig. 4:**
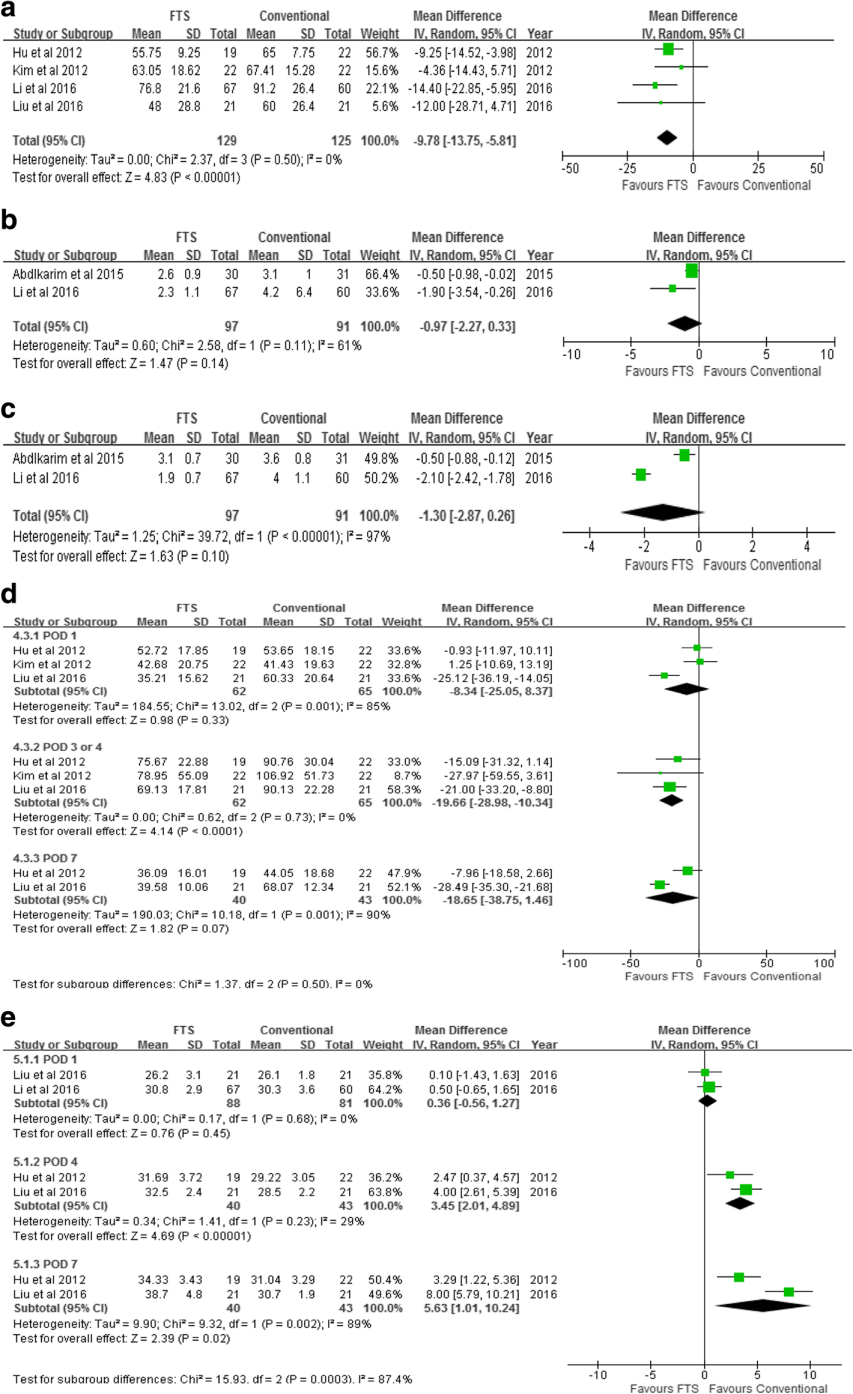
Meta-analyses of secondary outcomes. **a** Time to first flatus. **b** Ambulation time. **c** Time to start diet. **d** C-reaction protein. **e** Albumin

The results of this meta-analysis revealed that FTS group was associated with a shorter time to first flatus (WMD − 9.78; 95% CI − 13.75 to − 5.81, *P* < 0.00001) (Fig. 4a). No significant differences were found between the two groups in terms of ambulation time (WMD − 0.97; 95% CI − 2.27 to 0.33, *P* = 0.14) (Fig. 4b) and time to start diet (WMD − 1.30; 95% CI − 2.87 to 0.26, *P* = 0.10) (Fig. 4c).

#### C-reaction protein, albumin, and Interleukin-6

Three studies reported the C-reaction protein level on postoperative day 1. There was no significant difference between the two groups (WMD = − 8.34; 95% CI − 25.05 to 8.37, *P* = 0.33) (Fig. 4d). Results suggested that FTS group was associated with a lower level of C-reaction protein on postoperative days (PODs) 3 and 4 (WMD − 19.66; 95% CI − 28.98 to − 10.34, *P* < 0.00001), and there was no significant difference between the two groups on postoperative day 7 (WMD − 18.65; 95% CI − 38.75 to 1.46, *P* = 0.07) (Fig. 4d). Two studies reported the albumin level on postoperative day 1. No significant difference was observed between the two groups (WMD = 0.36; 95% CI − 0.56 to 1.27, *P* = 0.45) (Fig. 4e). However, the results showed that FTS group was associated with a higher level of albumin on postoperative day 4 (WMD 3.45; 95% CI 2.01 to 4.89, *P* < 0.00001) and postoperative day 7 (WMD 5.63; 95% CI 1.01 to 10.24, *P* = 0.02) (Fig. 4e).

## Discussion

ERAS was first proposed by the Danish physician Kehlet, with the aim of reducing surgical trauma and facilitating postoperative recovery by the use of a series of perioperative management [5]. Nowadays, laparoscopic surgery have garnered tremendous popularity over open surgery with better short-term outcomes, such as less operative bleeding, earlier bowel movement, reduced pain, fewer overall complications, and shorter postoperative hospital stays [25–27]. To date, ERAS and laparoscopic technique have been widely applied. However, the benefit of ERAS in patients undergoing laparoscopic gastrectomy is still unclear. To the best of our knowledge, this is the first meta-analysis focus on this topic. Comparing with previous meta-analyses mainly focus on open gastrectomy, the surgical procedure of our study was limited to LAG.

The results of the meta-analysis suggest that the FTS group is associated with a significant reduction in postoperative hospital stay, time to first flatus, postoperative CRP, IL-6, and hospital charge as compared with conventional care group. Additionally, no difference in postoperative complications and readmission rate was observed when comparing ERAS and conventional care within LAG.

A shorter hospital stay was the advantage of laparoscopic surgery. Our results showed that ERAS combined with LAG could shorten the length of postoperative hospital stay as compared with conventional care. Meanwhile, we also found that all included studies showed a consistent tendency favoring the FTS group. A previous meta-analysis suggested that ERAS combined with LAG are associated with a significant reduction in postoperative hospital stay of 2.68 days as compared with conventional care. Our meta-analysis showed that FTS group was associated with a reduction in postoperative hospital stay of 2.16 days. Secondly, we found in the present study that FTS group are associated with a significant reduction in time to first flatus. Four included studies with appropriate data that reported this outcome showed a result favoring FTS group. It has been widely accepted that both LAG and ERAS can reduce surgical trauma and facilitating postoperative recovery. Our results revealed that the combined effects of the two methods can further accelerate clinical recovery of the patients undergoing LAG.

Postoperative complication is the key indicator for assessing the safety and feasibility of surgical procedure. Extensive studies have shown that LAG is associated with fewer complications, such as incision infection and pneumonia as compared with OG. Meanwhile, one of the main principles of the ERAS protocol is reducing postoperative complications. Previous meta-analyses and RCTs showed reduced postoperative complications and readmission rate when ERAS was implemented in OG [14, 16, 28–30]. A previous study in our department demonstrated that the postoperative complication rate in the FTS group was lower than that in the conventional care group. They found that the ERAS protocol could reduce the incidence of pneumonia. This benefit may mainly attribute to the early ambulation of patients [30]. Li et al. [24] also reported that the FTS group was associated with a reduction in postoperative complication rate following LAG. Our result also showed a similar tendency favoring FTS, but there was no significant difference between the two groups. The statistical insignificance may be attributed to the reduction of some common complications such as incision infection, and pneumonia may have already been achieved by LAG, leaving little room for improvement via the implement of ERAS protocol. Additionally, it may also due to the relative small sample size of the present study.

The use of abdominal drains following gastrectomy still remains controversial. Prophylactic peritoneal drainage has been widely used during gastrointestinal surgery because of several advantages such as removing intraperitoneal fluid and assisting the early detection of postoperative hemorrhage or anastomotic leakage [31–33]. However, peritoneal drainage can cause uncomfortable which may limit early mobilization and postoperative recovery. There is evidence that abdominal drains do not reduce the complication rate but increase intraperitoneal fluid collection, infections, and risk of occurrence of postoperative fistula [34]. Despite of these disadvantages, peritoneal drainage is still commonly used for gastric cancer surgery at most institutions. Only one of the included studies in this meta-analysis did not use peritoneal drainage as routine treatment [18]. Thus, the safety and efficacy of no routine use of abdominal drainage tube need to be assessed in further investigation.

It have been recognized widely that the advantages of laparoscopic surgery such as alleviating surgical stress and reducing respiratory interference could accelerate postoperative recovery [35–37]. To date, some authors have reported that ERAS protocol could accelerate recovery by mitigating the inflammatory response [14, 15, 38, 39]. Our results suggest the combination of the two methods can further alleviate the inflammation and immune inhibition based on the efficacy of a single method. Liu et al. [40] indicated that FTS group showed lower C-reaction protein and IL-6 levels (postoperative days 1, 4, and 7) compared with the conventional care group. The level of serum albumin is a nutritional status indicator. Our results showed that the albumin in the FTS group was higher than that in the conventional perioperative care group on postoperative days 4 and 7. We presumed that this difference may mainly attributed to the early enteral nutrition (EN) improved postoperative nutrition status in FTS group. In addition, several meta-analyses have demonstrated that early EN may decrease early occurrence of postoperative infections, shorten the length of hospital stay and therefore promote the postoperative recovery of paitents [41–45].

Researches have shown that laparoscopic surgery is associated with better quality of life (QOL) as compared with open surgery. Regarding the QOL following ERAS combined with LAG, studies have been seldom reported. Kim et al. [17] reported that the ERAS protocol may have no negative effect on QOL or patient satisfaction. Moreover, certain aspects of QOL such as pain, fatigue, appetite loss, and financial problems can be improved by the implement of ERAS protocol. Our study has several limitations. First, the included studies are all from Aisa and in relatively small sample size, which may limited the generalization of our results. Second, the differences in patient characteristics is a source of bias that may affect the stability of the results. Third, although time to first flatus had low heterogeneity, other outcomes had moderate or high heterogeneity. Moreover, the compliance is varied among all included studies which may reduce the benefits of ERAS protocol.

## Conclusions

In conclusion, this meta-analysis indicates that ERAS combined with laparoscopic technique is safe and effective for gastric cancer and could significantly decrease postoperative hospital stay, cost of hospitalization, and time to first flatus without increasing postoperative complication and readmission rate. High-quality and larger-scale studies are needed to provide more solid evidence.
